# Sex Differences in Sleep Health and Aging

**DOI:** 10.1093/geroni/igaf122.486

**Published:** 2025-12-31

**Authors:** Ashley Curtis, Christopher Kaufmann, Christopher Kaufmann

## Abstract

Growing evidence highlights significant sex differences in sleep patterns, disorders, and their health consequences. Given well-documented age-related changes in sleep patterns overall, there is a need to study sex differences in sleep and associated outcomes in mid-to-late life. This symposium will showcase emerging research in this field and offer insights into sex-specific sleep and circadian patterns, as well as the differential effects on cardiovascular, neurocognitive, and physical health. Paper 1 will characterize sex differences in multidimensional sleep health using data from the Midlife in the United States study (MIDUS). The Ru-SATED sleep health framework proposed by Dr. Buysee will guide examinations of cross-sectional and longitudinal sex differences in core sleep health components (e.g., regularity, timing, efficiency, duration). Paper 2 will examine sex differences in circadian patterns. Using MIDUS, we will present key sex differences in actigraphy-assessed Rest-Activity Rhythms patterns, and their impact on cardiometabolic factors (e.g., HbA1C, total cholesterol). Paper 3 will describe sex-specific patterns of association between sleep health, cardiovascular risk profiles and cognition in mid-to-late life. Paper 4 will present data from the Thinking and Living with Cancer study comparing effects of poor sleep health on physical functioning in older women with breast cancer versus controls. As Discussant, Dr. Stone will integrate findings and discuss future research directions. Overall, our symposium provides unique insight into sex differences in sleep health and their impact on physical, cardiovascular and neurocognitive functioning. This session can guide future treatments for sleep disturbances, promoting successful aging in both men and women.

